# Socially Anxious Tendencies Affect Impressions of Others’ Positive and Negative Emotional Gazes

**DOI:** 10.3389/fpsyg.2018.02111

**Published:** 2018-11-01

**Authors:** Yuki Tsuji, Sotaro Shimada

**Affiliations:** ^1^Department of Electronics and Bioinformatics, School of Science and Technology, Meiji University, Kawasaki, Japan; ^2^Japan Society for the Promotion of Science, Tokyo, Japan

**Keywords:** social anxiety disorder, gaze perception, emotional gazes, impression, morphing

## Abstract

Socially anxious tendencies have potential to become social anxiety disorder (SAD), which is characterized by fear of social situations associated with being evaluated or embarrassed by others. In particular, others’ gazes induce social anxiety. People with SAD have a negative interpretation bias toward ambiguous emotions in others’ faces; however, negative interpretation bias toward ambiguous emotions in others’ gazes has not been fully investigated. We used an impression judgment task to examine negative interpretation bias toward others’ gazes among people with socially anxious tendencies. We generated emotionally ambiguous gazes (positive, negative, and neutral) using a morphing technique with 10% steps (neutral, 10–100% negative, and 10–100% positive). Participants (all male) were asked to judge whether the stimulus was positive or negative. Each participant’s level of social anxiety was examined using the Japanese version of the Social Phobia Inventory (SPIN-J), which measures three symptom dimensions: fear, avoidance, and physiological arousal. To examine the influence of socially anxious tendencies in the impression judgment task, we calculated the point of subjective equality (PSE) using a two-step logistic curve fitted to individual participant’s responses. The negative emotional intensity of the PSE became lower as the fear score became higher (*p* < 0.05). This result suggests individuals with a high tendency toward social anxiety tend to interpret subtle negative emotional gazes as a negative emotion and regard these gazes as a threat.

## Introduction

To guess emotion is one of important abilities in social interaction. Humans are able to appropriately judge emotions from facial expressions, with this ability extending beyond cultural boundaries (Darwin, 1872; Ekman et al., 1969). However, impressions received from facial expressions vary by individual characteristics. The constructed emotion theory suggests that categorizing one’s own or others’ emotions depends on integrating information from the inside world (e.g., interoception, memorized or imagined representations, and concepts) and the outside world (e.g., five senses) to obtain meaning (Russell, 2003; Barrett et al., 2007; Barrett, 2012). According to this theory, suitably guessing others’ emotion from others’ facial expressions requires the categorized emotion and the other person’s emotion to correspond. Cognitive models of social anxiety suggest that socially anxious individuals have an attentional bias for negative social cues that could indicate social rejection or threat (Rapee and Heimberg, 1997). In social situations, individuals with high socially anxious (HSA) tendencies tend to pay attention to themselves as social objects rather than focusing on people around them (Clark and Wells, 1995). This bias is thought to activate negative self-beliefs (e.g., “others dislike me”). These theories and cognitive models suggest individuals with social anxiety tend to use their inside world information as clues for judging emotions, thereby making it difficult to suitably guess others’ emotions. A defining feature of social anxiety disorder (SAD) or social phobia is avoidance or excessive fear of situations associated with evaluation or embarrassment by others (American Psychiatric Association [APA], 2000). Some studies reported that individuals with HSA tendencies or SAD were more likely to evaluate or misinterpret facial expressions as threatening (Dimberg et al., 1986; Pozo et al., 1991; Winton et al., 1995; Dimberg, 1997; Heuer et al., 2010; Vassilopoulos, 2011). In particular, clinical studies indicate that patients with SAD recognized others’ facial expressions as a threat (Heinrichs and Hofmann, 2001; Hirsch and Clark, 2004).

Several studies have used Likert-type self-report scales to rate the perceived intensity of facial emotional expressions during neuroimaging or following experimental procedures as a control condition (for a review see Staugaard, 2010). However, studies using morphing techniques to generate faces expressing varying intensities of emotion provide inconsistent behavioral evidence for the effect of social anxiety on facial emotion identification. For example, Joormann and Gotlib (2006) reported that individuals with HSA tendencies had a lower threshold for identifying angry faces relative to healthy participants. In contrast, Montagne et al. (2006) reported that healthy participants had a lower threshold for identifying angry faces relative to those with HSA tendencies. However, individuals with HSA rate negative emotional facial expressions as more negative than individuals with low socially anxious (LSA) tendencies or healthy participants (Dimberg and Christmanson, 1991; Dimberg and Thunberg, 2007; Schofield et al., 2007; Goldin et al., 2009). Furmark et al. (2009) reported that participants with social phobia showed more anxiety responses to angry or neutral faces than healthy controls. Other studies reported no association between social anxiety and identifying facial expressions (Philippot and Douilliez, 2005; Schofield et al., 2007).

Individuals with high trait anxiety tend to classify blended angry and disgusted expressions as disgusted expressions (Richards et al., 2002). Socially anxious individuals fear being evaluated as incompetent or disgusting more than they fear provoking others’ anger (American Psychiatric Association [APA], 2000). Cognitive models of social anxiety suggest that socially anxious individuals activate negative self-beliefs (e.g., incompetent or disgust) in response to perceived social threats (Clark and Wells, 1995; Rapee and Heimberg, 1997). Individuals with HSA also tend to rate disgusted faces as more negative than angry faces (Amir et al., 2010). Therefore, others’ disgusted expressions may induce activity of negative self-beliefs in individuals with socially anxious tendencies. Patients with SAD also confused ambiguous or neutral emotional expressions with negative emotions (e.g., anger, threat, disgust) relative to healthy people (Bell et al., 2011). These findings suggest that individuals with HSA have a negative interpretation bias toward disgusted, ambiguous, and happy facial expressions relative to those with LSA.

Mathews et al. (2003) reported that participants with high trait anxiety showed enhanced orienting to the gaze cued location of faces with fearful expressions, relative to other expressions. Similar effects have been found when comparing high and low state anxiety (Holmes et al., 2006). Anxiety is associated with enhanced attentional cuing by fearful eye gazes (Fox et al., 2007), and social anxiety is associated with increased orientation to facial threats (Mogg and Bradley, 2002; Mogg et al., 2007) and aversion to direct eye gaze (Schulze et al., 2013a). The perception of direct gaze also varies as a function of trait anxiety, with a bias toward perceiving slightly averted gazes as direct gazes in clinically and non-clinically anxious individuals (Schulze et al., 2013a,b). Despite the fact that the gazes of other people commonly induce social anxiety (Den Boer, 2000), the way in which people with SAD interpret others’ emotional gazes has not been fully investigated.

The present study examined the influence of socially anxious tendencies on subjective impressions of emotional gazes. We investigated responses or impressions to positive or negative emotional gazes of varying emotional intensities using morphing techniques. Previous reports indicated that individuals with clinical or subclinical social anxiety rated negative emotional facial expressions, especially disgusted faces (Amir et al., 2010), as more negative (Dimberg and Christmanson, 1991; Dimberg and Thunberg, 2007; Schofield et al., 2007; Goldin et al., 2009). These individuals also classified ambiguous emotional facial expressions as negative (Melfsen and Florin, 2002; Bell et al., 2011), and rated positive emotional facial expressions as less pleasant (Straube et al., 2004). Therefore, we hypothesized that socially anxious tendencies would modulate subjective impressions of ambiguity of others’ emotional gazes (disgusted or happy). We assumed that using a Likert scale to respond to the impression of the stimulus might allow ambiguous answers, and would not clarify the influence of socially anxious tendencies on subjective impressions of emotional gazes. Therefore, we used a forced two-choice task (impression judgment task) to clarify this issue.

## Materials and Methods

### Participants

McLean et al. (2011) reported that there were no significantly difference of the lifetime and 12-month prevalence rates of SADs across gender. We assumed that there was no difference of impressions response to emotional gazes across gender. We collected data from male participants to match the gender of participants and that of stimuli. Participants were 32 healthy male volunteers (mean age 21.4 ± 1.21 years). All participants had normal or corrected-to-normal vision, and provided written informed consent to participate in this study. The study protocol was approved by the Ethics Committee of the School of Science and Technology, Meiji University. This study was conducted according to the principles and guidelines of the Declaration of Helsinki.

### Social Anxiety Rating

Each participant’s level of social anxiety was measured with the 17-item Japanese version of the Social Phobia Inventory (SPIN-J). Each item is rated on a 5-point Likert-type scale, giving a total score of 0–68. The SPIN-J measures three symptom dimensions: fear, avoidance, and physiological arousal (Connor et al., 2000). The SPIN-J is unique in that it contains a physiological subscale. This subscale may be particularly important in Japan because East Asian patients with anxiety disorders tend to somaticize their symptoms (Kirmayer, 2001). The fear and avoidance scale displayed a good internal consistency (Cronbach’s alpha = 0.83,0.81, respectively), but the arousal scale displayed a poor internal consistency (Cronbach’s alpha = 0.27) in the present sample.

### Gaze Stimuli

The experimental stimuli were grayscale images of the human eye region. We generated prototypical emotional (happy and disgusted) and neutral gazes. These prototypes were produced from pictures the eyes of four Japanese male volunteers (mean age 23.0 ± 1.15 years) using Adobe Photoshop CS6.0 software. We used eye region of natural smile as positive emotional gazes, that of disgusted face as negative emotional gazes and that of neutral face as neutral gazes, respectively. When we took a picture of each actor’s neutral face, we instructed them to keep as expressionless as possible. We took a picture of the actors’ natural smiles, when they began to spontaneous laughter. To take a picture of each actor’s disgusted face, we asked them to think of an aversive episode. Independent raters, who were 15 male volunteers (mean age 22.0 ± 2.42 years), chose among six emotions (happiness, sadness, anger, disgust, surprise, fear) to describe happy gazes and disgusted gazes and they classified these gazes into positive or negative emotion. The happy gazes were the most frequently judged as happiness (57.0 ± 6.20%) and also chosen as a positive emotion (60.0 ± 6.36%). The disgusted gazes were the most frequently judged as anger (56.7 ± 6.67%), the second frequently judged as disgust (30.0 ± 6.55%), and also chosen as a negative emotion (100 ± 0.00%). Thus, we were confident that experimental stimuli were interpreted as a negative or positive stimulus.

The images occupied 3.4° × 13.4° of the visual field (4.7 × 19 cm). We morphed each emotion prototype with a neutral image using 16 reference points (three points at equal intervals on the upper and lower eyelids, one point at the inner corners of the eyes and one point at the outer canthus) to generate continua with 100 emotion intensities. We selected 10 intensities for each emotion (10%–100% in 10% steps), giving a total of 84 images. The stimuli were presented at the center of a 27-inch LCD monitor using E-Prime software (Psychology Software Tools, PA, United States), and viewed at a distance of approximately 80 cm. We displayed part of the experimental stimuli (each volunteer’s 100% positive, neutral, and 100% negative gazes) before beginning the experiment. Participants were instructed that these stimuli showed a full smile, expressionlessness, and visible distaste, respectively. The 84 stimuli are provided as Supplementary Material.

### Procedure

**FIGURE 1:**
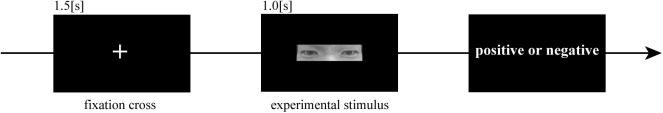
Timeline of the trial. The experimental stimulus was displayed for 1.0 s, after which a fixation cross appeared for 1.5 s. The next trial began after participants provided their answers.

In each trial, the experimental stimulus was displayed for 1.0 s. Participants were instructed to fixate on the eye region of the stimulus during stimulus presentation, and indicate their impression of the stimulus as negative or positive, corresponding to emotional intensities for positive or negative (impression judgment task). After participants provided their answers, a fixation cross was displayed for 1.5 s and then the next trial was initiated (Figure 1). An experimental session consisted of 84 trials. Each session for each participant lasted approximately 5 min, and each participant underwent four experimental sessions. All 84 gaze stimuli were presented in each session, giving a total of 336 trials over the four sessions. The order of stimulus presentation was random in each session.

### Data Analysis

Participants were asked to judge whether their impression of each experimental stimulus was positive or negative. We plotted participants’ mean responses using graphs, with the negative response rate as the vertical axis and the emotional intensity of the experimental stimulus as the horizontal axis. We observed a plateau around neutral.

**FIGURE 2:**
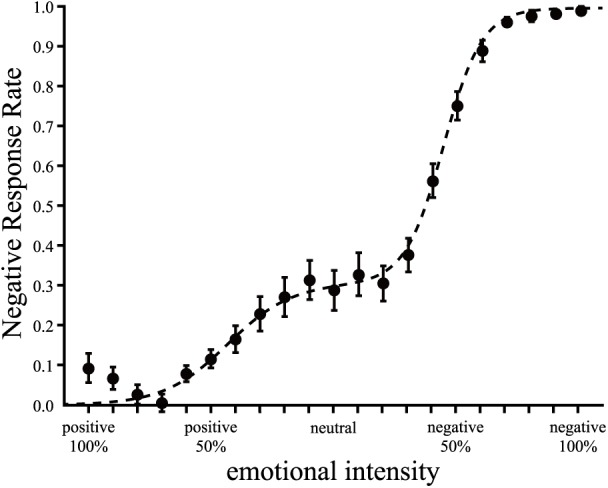
Mean negative response rate for all participants and the fitted curves. The black circles represent the mean responses of participants each emotional intensity level. The broken line represents negative judgment curve which fitted to mean negative response for all participants. Error bars represent the standard error.

To examine the influence of socially anxious tendencies in judgment, we fitted non-linear regression curves to individual participant responses. We used two models of curve fit as a preliminary analysis: a logistic curve and a two-step logistic curve. The logistic curve represented the entire shape of participants’ responses, whereas the two-step logistic curve represented the entire shape of participants’ responses and a plateau (Figure 2). We estimated goodness of fit for each model by calculating the Akaike Information Criterion (AIC) (Akaike, 1998) and Bayesian Information Criterion (BIC) (Schwarz, 1978). The AIC and BIC for the two-step logistic curve fitted to individual participant’s responses were smaller than when the logistic curve was fitted. The results of curve fit using the two-step logistic curve model showed that goodness of fit was better relative to the other model. Therefore, we selected the two-step logistic curve model using the following formula

$$P(x)=\frac{a}{1+exp\{-b(x-c)\}}+\frac{1-a}{1+exp\{-d(x-e)\}}.$$

The first term represents the sigmoid shape on the negative emotional side. The second term represents the sigmoid shape on the positive emotional side. In the first term, *x* is the emotional intensity, *P(x)* the probability of negative judgment, *a* the rate of negative judgment of negative emotional gazes, *b* the steepness of the fitted curve, and *c* the inflection point. In the second term, 1 - *a* indicates the rate of negative judgment of ambiguous emotional gazes, *d* the steepness of the fitted curve, and *e* the inflection point. Curve fit was performed using a non-linear least squares method (a trust-region algorithm), provided by the Curve Fitting Toolbox in MATLAB R2015b (The MathWorks Inc., Natick, MA, United States). We used Spearman’s rank correlation coefficients to examine correlations between SPIN-J scores and the point of subjective equality (PSE), and between SPIN-J scores and the parameter of the curve fit model. To control for type 1 error, Bonferroni correction was applied for correlation analyses.

## Results

Figure 2 shows the means of participants’ responses and the fitted curve.

**FIGURE 3:**
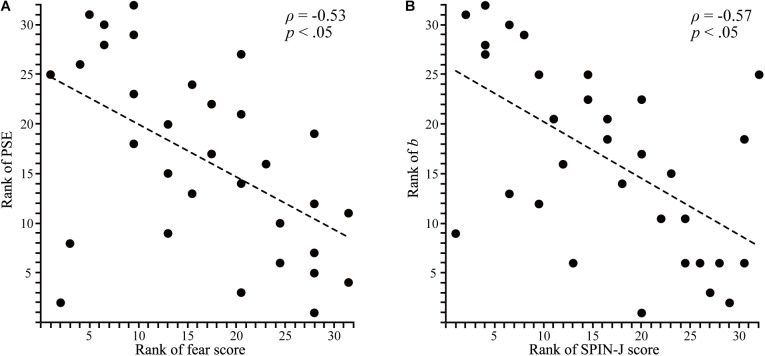
Correlations between fear score rank and point of subjective equality rank, and SPIN-J score rank and *b* rank. **(A)** Regression line for a negative correlation between the point of subjective equality rank and fear score rank. The Spearman’s rank correlation coefficient was ρ = -0.53 (*p* < 0.05). **(B)** Regression line for a negative correlation between *b* rank and SPIN-J score rank. The Spearman’s rank correlation coefficient was ρ = -0.57 (*p* < 0.05).

We found a significant negative correlation between the fear score (SPIN-J subscale) and PSE (*ρ* = -0.53, *p* < 0.05; Figure 3A). This indicated that the intensity of negative emotion giving a negative impression became lower as the fear score became higher. We found significant negative correlations between *b* (which indicates the sigmoid curve’s slope at the negative emotional side) and the SPIN-J score (*ρ* = -0.57, *p* < 0.05; Figure 3B) and fear score (*ρ* = -0.53, *p* < 0.05). This indicated that the sigmoid curve’s slope at the negative emotional side became gentler as the SPIN-J score (especially fear score) became higher. There were no significant correlations between other SPIN-J subscales and PSE or other parameters of the fitted curve.

## Discussion

The present study examined the influence of socially anxious tendencies on impressions of others’ positive and negative emotional gazes. The present results showed that the negative emotional intensity that was regarded as a negative impression became lower as the SPIN-J fear subscale score became higher. Button et al. (2013) performed an expression classification task in which the experimental stimuli were facial expressions that were changed using morphing technology; participants with HSA tendencies more often misclassified emotional expressions than those with LSA tendencies. A previous study reported that children with socially anxious tendencies classified neutral faces as emotional faces (Melfsen and Florin, 2002). Another study reported that patients with SAD more often classified neutral faces as angry faces than healthy people (Bell et al., 2011). Consistent with previous studies, our results suggest that individuals with a high tendency toward social anxiety interpreted subtle intensities of negative gazes as negative expressions.

The amygdala activation related with social threat perception in individuals with HSA tendencies. A positron emission tomography study reported that the amygdala response to public speaking decreased following cognitive behavioral therapy or administration of selective serotonin reuptake inhibitors (Furmark et al., 2002). Selective serotonin reuptake inhibitors are the most commonly prescribed class of drugs for depression, anxiety, and obsessive-compulsive disorder (Lockhart and Guthrie, 2011). Some functional magnetic resonance imaging (fMRI) studies have suggested that increased activity of the amygdala is related to the degree of social anxiety (Straube et al., 2004; Cooney et al., 2006; Stein and Stein, 2008). People with SAD showed increased activation of the amygdala more often in response to angry expressions than to neutral expressions. In those with SAD, activation of the amygdala response to angry faces also increased compared with the response to neutral faces (Straube et al., 2004). Some studies have reported that the amygdala has a role in increasing responses to fearful faces (Whalen et al., 1998; Liddell et al., 2005), and amygdala activation is assumed to be related to social threats, such as fearful gazes (Kanat et al., 2015). Individuals with SAD showed increased amygdala activity in response to neutral or ambiguous emotional faces compared with healthy people (Cooney et al., 2006). The activity of the amygdala in response to emotional faces suggested that individuals with SAD regarded emotional faces as socially threatening. Our results, which showed that the negative emotional intensity (which was regarded as the negative impression) became lower as the SPIN-J fear subscale score became higher, suggested that individuals with a high tendency toward social anxiety recognized negative emotional gazes of subtle intensity as social threats. In addition, our results showed that the sigmoid curve’s slope at negative emotional side gentled as the SPIN-J score (especially fear score) became higher. Some studies suggested that individuals with HSA tendencies attribute excessive psychological cost to disgusted, angry, sad, or neutral faces (Schofield et al., 2007; Douilliez et al., 2012; Button et al., 2013). Our results also indicated that socially anxious tendencies increased negative impressions of negative emotional gazes.

There was the bump on the positive emotional side (left side in Figure 2) in the mean negative response rates. The independent raters classified happy gazes into various emotions. Several studies have reported associations between a smile and various emotions other than positive emotions (Niedenthal et al., 2010; Ambadar et al., 2009; Calvo et al., 2013a,b). For example, a smile may be associated with negative emotions such as social dominance, irony, ridicule, or embarrassment. The bump on the positive emotional side suggested that 100% positive emotional gazes were associated with other negative emotions. Although some studies reported associations between positive emotions and negative impressions, our results showed no significant correlation between the SPIN-J scores and the parameters of sigmoid shape on the positive emotional side. Other studies suggested that although individuals with HSA tendencies attributed excessive psychological cost to negative or neutral faces, such excessive psychological cost was not attributed to happy faces to the same extent (Schofield et al., 2007; Button et al., 2013). Consistent with these findings, our results suggested that individuals with a high tendency toward social anxiety had no excessive psychological cost attributed to positive emotional gazes compared with negative emotional gazes.

The present results suggest that others’ ambiguous emotional gazes induced more anxiety in individuals with a high tendency toward social anxiety; consequently, individuals with a high tendency toward social anxiety perceived these emotional gazes as threatening. Some fMRI studies have shown that neutral, ambiguous, or negative facial expressions can induce hyperactivation of the amygdala in individuals with SAD (Cooney et al., 2006; Blair et al., 2008; Labuschagne et al., 2010). Further studies of amygdala function would complement the present findings, and increase our understanding of negative interpretation bias in individuals with socially anxious tendencies toward others’ ambiguous emotional gazes.

## Conclusion

This study aimed to reveal the effects of socially anxious tendencies on impressions of others’ positive and negative emotional gazes. We found that the negative emotional intensity that was regarded as a negative impression became lower as the socially anxious tendencies became higher. Therefore, we suggest that negative emotional gazes of subtle intensity may induce social anxiety in individuals with a high tendency toward social anxiety. Individuals with a high tendency toward social anxiety are more likely to have a negative interpretative bias toward negative emotional gazes of even subtle intensity, and interpret these subtle intensity negative emotional gazes as threats.

## Author Contributions

YT and SS designed the experiments and wrote the manuscript. YT performed the experiments, collected the data, and analyzed the data.

## Conflict of Interest Statement

The authors declare that the research was conducted in the absence of any commercial or financial relationships that could be construed as a potential conflict of interest.
