# Supplementary material for: Socially Anxious Tendencies Affect Impressions of Others’ Positive and Negative Emotional Gazes
Source: Front Psychol. 2018 Nov 1;9:2111. doi: 10.3389/fpsyg.2018.02111 (PMC6221960; doi:10.3389/fpsyg.2018.02111)
Supplement: Supplementary file 1 [file Table_1.DOCX]

Supplementary Material

Socially Anxious Tendencies Affect Impressions of Others’ Happy and Disgusted Gazes

Yuki Tsuji, Sotaro Shimada*

*** Correspondence:** Sotaro Shimada: sshimada@meiji.ac.jp

## Supplementary Table1. Happy stimuli increasing in intensity 0% to 100% top to bottom in 10% increments.

| neutral | neutral | neutral | neutral |
| --- | --- | --- | --- |
| 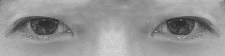 | 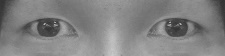 | 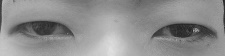 | 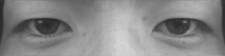 |
| 10% happy | 10% happy | 10% happy | 10% happy |
| 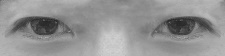 | 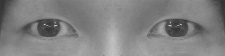 | 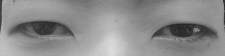 | 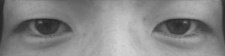 |
| 20% happy | 20% happy | 20% happy | 20% happy |
| 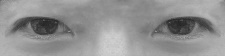 | 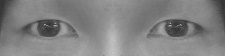 | 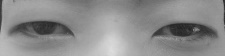 | 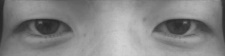 |
| 30% happy | 30% happy | 30% happy | 30% happy |
| 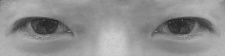 | 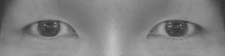 | 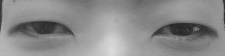 | 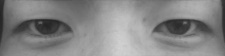 |
| 40% happy | 40% happy | 40% happy | 40% happy |
| 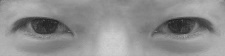 | 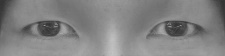 | 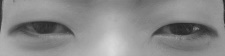 | 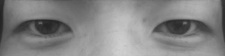 |
| 50% happy | 50% happy | 50% happy | 50% happy |
| 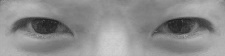 | 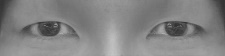 | 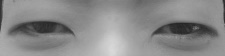 | 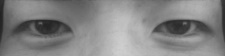 |
| 60% happy | 60% happy | 60% happy | 60% happy |
| 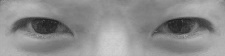 | 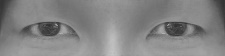 | 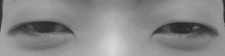 | 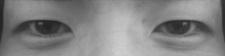 |
| 70% happy | 70% happy | 70% happy | 70% happy |
| 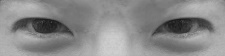 | 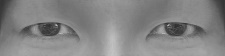 | 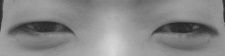 | 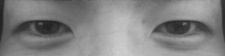 |
| 80% happy | 80% happy | 80% happy | 80% happy |
| 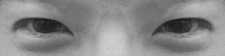 | 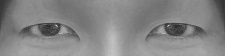 | 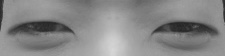 | 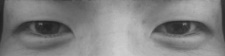 |
| 90% happy | 90% happy | 90% happy | 90% happy |
| 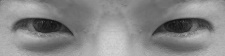 | 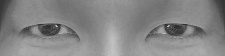 | 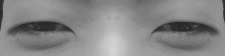 | 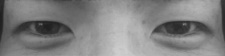 |
| 100% happy | 100% happy | 100% happy | 100% happy |
| 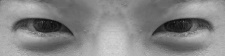 | 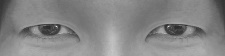 | 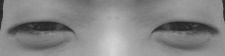 | 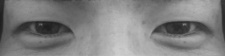 |
