# Supplementary material for: Socially Anxious Tendencies Affect Impressions of Others’ Positive and Negative Emotional Gazes
Source: Front Psychol. 2018 Nov 1;9:2111. doi: 10.3389/fpsyg.2018.02111 (PMC6221960; doi:10.3389/fpsyg.2018.02111)
Supplement: Supplementary file 2 [file Table_2.DOCX]

Supplementary Material

Socially Anxious Tendencies Affect Impressions of Others’ Happy and Disgusted Gazes

Yuki Tsuji, Sotaro Shimada*

*** Correspondence:** Sotaro Shimada: sshimada@meiji.ac.jp

## Supplementary Table2. Disgusted stimuli increasing in intensity 0% to 100% top to bottom in 10% increments.

| neutral | neutral | neutral | neutral |
| --- | --- | --- | --- |
| 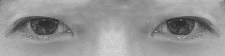 | 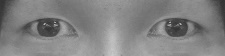 | 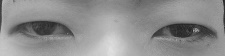 | 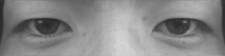 |
| 10% disgust | 10% disgust | 10% disgust | 10% disgust |
| 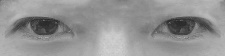 | 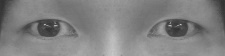 | 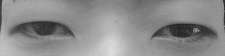 | 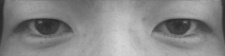 |
| 20% disgust | 20% disgust | 20% disgust | 20% disgust |
| 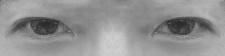 | 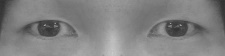 | 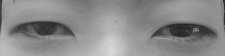 | 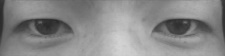 |
| 30% disgust | 30% disgust | 30% disgust | 30% disgust |
| 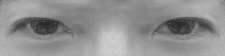 | 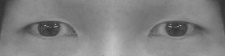 | 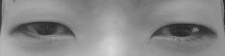 | 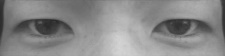 |
| 40% disgust | 40% disgust | 40% disgust | 40% disgust |
| 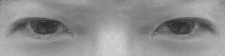 | 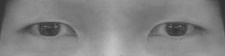 | 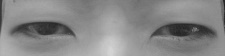 | 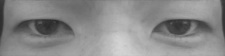 |
| 50% disgust | 50% disgust | 50% disgust | 50% disgust |
| 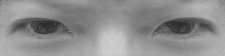 | 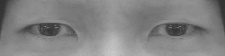 | 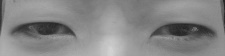 | 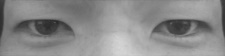 |
| 60% disgust | 60% disgust | 60% disgust | 60% disgust |
| 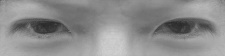 | 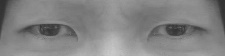 | 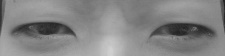 | 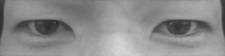 |
| 70% disgust | 70% disgust | 70% disgust | 70% disgust |
| 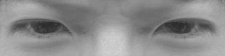 | 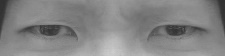 | 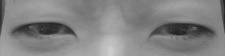 | 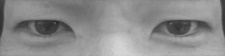 |
| 80% disgust | 80% disgust | 80% disgust | 80% disgust |
| 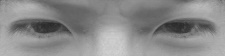 | 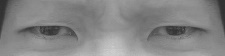 | 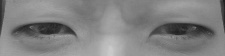 | 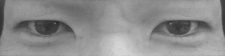 |
| 90% disgust | 90% disgust | 90% disgust | 90% disgust |
| 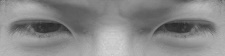 | 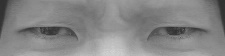 | 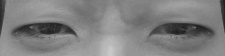 | 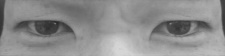 |
| 100% disgust | 100% disgust | 100% disgust | 100% disgust |
| 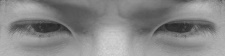 | 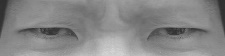 | 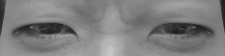 | 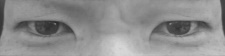 |
